# The Cerebellar Nodulus/Uvula Integrates Otolith Signals for the Translational Vestibulo-Ocular Reflex

**DOI:** 10.1371/journal.pone.0013981

**Published:** 2010-11-15

**Authors:** Mark F. Walker, Jing Tian, Xiaoyan Shan, Rafael J. Tamargo, Howard Ying, David S. Zee

**Affiliations:** 1 Department of Neurology, The Johns Hopkins University School of Medicine, Baltimore, Maryland, United States of America; 2 Department of Ophthalmology, The Johns Hopkins University School of Medicine, Baltimore, Maryland, United States of America; 3 Department of Neurosurgery, The Johns Hopkins University School of Medicine, Baltimore, Maryland, United States of America; 4 Department of Otolaryngology-Head and Neck Surgery, The Johns Hopkins University School of Medicine, Baltimore, Maryland, United States of America; 5 Department of Neuroscience, The Johns Hopkins University School of Medicine, Baltimore, Maryland, United States of America; 6 Department of Neurology, Case Western Reserve University and Veterans Affairs Medical Center, Cleveland, Ohio, United States of America; Claremont Colleges, United States of America

## Abstract

**Background:**

The otolith-driven translational vestibulo-ocular reflex (tVOR) generates compensatory eye movements to linear head accelerations. Studies in humans indicate that the cerebellum plays a critical role in the neural control of the tVOR, but little is known about mechanisms of this control or the functions of specific cerebellar structures. Here, we chose to investigate the contribution of the nodulus and uvula, which have been shown by prior studies to be involved in the processing of otolith signals in other contexts.

**Methodology/Principal Findings:**

We recorded eye movements in two rhesus monkeys during steps of linear motion along the interaural axis before and after surgical lesions of the cerebellar uvula and nodulus. The lesions strikingly reduced eye velocity during constant-velocity motion but had only a small effect on the response to initial head acceleration. We fit eye velocity to a linear combination of head acceleration and velocity and to a dynamic mathematical model of the tVOR that incorporated a specific integrator of head acceleration. Based on parameter optimization, the lesion decreased the gain of the pathway containing this new integrator by 62%. The component of eye velocity that depended directly on head acceleration changed little (gain decrease of 13%). In a final set of simulations, we compared our data to the predictions of previous models of the tVOR, none of which could account for our experimental findings.

**Conclusions/ Significance:**

Our results provide new and important information regarding the neural control of the tVOR. Specifically, they point to a key role for the cerebellar nodulus and uvula in the mathematical integration of afferent linear head acceleration signals. This function is likely to be critical not only for the tVOR but also for the otolith-mediated reflexes that control posture and balance.

## Introduction

The otolith organs sense the head's linear motion and its orientation relative to gravity. Signals proportional to linear head acceleration are carried by otolith afferents from the labyrinth to the brain to drive the translational vestibulo-ocular reflex (tVOR), helping to stabilize vision when the head moves.

The central pathways of the tVOR are less well known than those of the rotational VOR (rVOR), although new information has emerged in recent years [1], [2]. Studies in humans with degenerative diseases suggest the cerebellum plays a role, since their response to interaural (lateral) translation is often dramatically attenuated [3]–[5]. In contrast, only a minority of cerebellar patients loses the rVOR [6], [7]; in most, the reflex is still present, although the amplitude, phase, and/or direction may be abnormal [8]–[11]. Which cerebellar substructures are important for the tVOR, and what their specific functions might be, is not known.

As part of the vestibulocerebellum, the nodulus and uvula (Nod/Uv) are densely connected to primary and secondary vestibular neurons, and they play a critical role in vestibular reflexes. The Nod/Uv receives a dense collateral projection from ipsilateral semicircular canal and otolith afferents. Saccular afferents project to the uvula [12], [13]; canal afferents project primarily to the nodulus [13]. Utricular afferents project to the uvula [12] in mice and to the nodulus in macaques [13]. Given this ambiguity, we chose to lesion both the nodulus and uvula in the present study.

Although the role of the Nod/Uv in the tVOR has only recently been considered, a number of studies have examined its contribution to the rVOR, including the interactions between canal and otolith inputs. The Nod/Uv plays a central role in angular velocity storage, the integrator of the central vestibular system that enhances the low-frequency performance of the rVOR [14]–[17]. Nod/Uv ablation also affects spatial orientation of the rVOR: it abolishes the normal reorientation of eye velocity to the gravito-inertial axis [18], [19]. Likewise, the roll rVOR is substantially reduced, and torsional OKN is impaired [20]. Recently, neural signals related to linear motion have been recorded in the Nod/Uv [21].

Using surgical lesions of the Nod/Uv in rhesus monkeys, we investigated for the first time whether these areas play a direct role in the horizontal tVOR. We measured eye velocity during abrupt steps of interaural translation; Nod/Uv lesions reduced the response to sustained translation but had much less effect on initial eye acceleration. Limited preliminary data from these experiments have been previously presented [22], [23]. Here we present new data and the results of detailed computational modeling to demonstrate that the Nod/Uv play an important role in the mathematical integration of head acceleration signals during translation.

## Results

### Responses to interaural translation in normal animals

**Figure 1:**
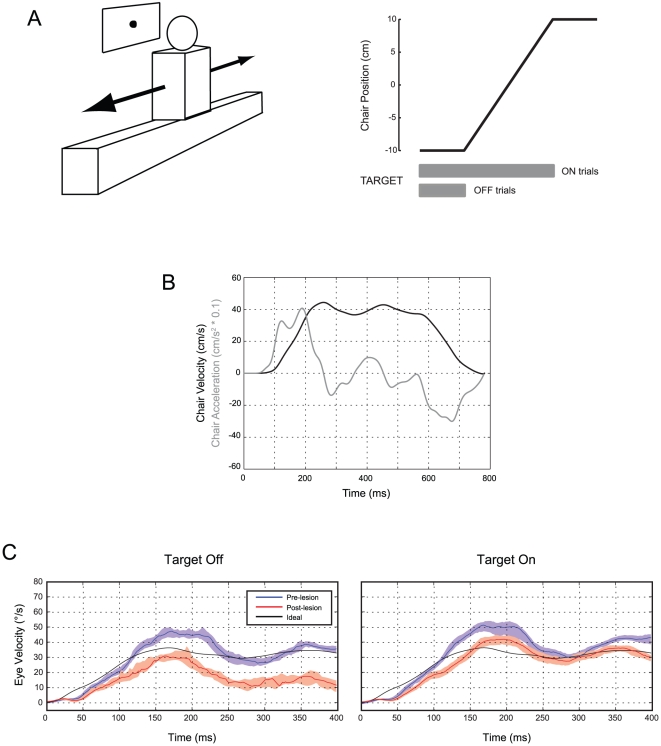
a. Schematic diagram of experimental setup (left) and a single motion trial (right). The monkey sat in a primate chair, facing a translucent screen on which was projected a laser target. The chair was translated along the interaural axis by a belt-driven motor. Each trial began with the monkey fixating the visual target located directly in front of its eyes. Then, the chair was translated at constant speed over a distance of 20 cm. For half of the trials, the target went out when the chair started to move; for the remainder, the target remained illuminated. After the chair stopped, the target was moved 20 cm, across from the new chair location, and the monkey fixated the target, starting a new trial. The chair moved back to the original position. **b**. Representative chair velocity and acceleration profiles (based on chair velocity feedback) for a single translation. Chair acceleration is scaled down by a factor of 10. **c**. Responses to rightward translation in animal M2, before and after lesioning of the Nod/Uv. The viewing distance is 70 cm. The solid colored lines indicate the median velocity across all trials (first 400 ms), excluding quick phases and saccades, and the shaded areas include the 25^th^ to 75^th^ velocity percentiles. Left panels show the responses when the target went off at the onset of chair movement, and right panels show the responses when the target remained on. The black line is the ideal eye velocity for this viewing distance. Data are plotted according to the right-hand-rule convention: leftward velocities are positive.

Figure 1 illustrates our experimental setup and shows the responses of M2 to abrupt rightward interaural translation when the target was at 70 cm. A robust and reproducible response was elicited, even when the target was extinguished at the onset of motion and translation occurred in darkness. Similar responses were recorded in M1 [22].

In both animals, there were two remarkable differences between the dynamics of the movement of the head and the movement of the eye. First, initial eye acceleration exceeded head acceleration. This partially compensated for the response latency, allowing the eyes to “catch up” with the head and reach their ideal velocities more quickly. Such behavior would not be expected if eye velocity were a linear function of head velocity alone. Second, during sustained chair motion, eye *velocity* oscillated about the ideal velocity. These oscillations appeared qualitatively similar to head *acceleration*, suggesting that eye velocity contains a signal component that is proportional to head acceleration. Thus, in our mathematical modeling (see below), we tested the hypothesis that eye velocity during interaural translation is derived from a combination of head velocity and head acceleration.

### Nod/Uv lesions reduce sustained eye velocity during steps of interaural translation

Ablation of the Nod/Uv reduced the tVOR, particularly in the dark (Figure 1C, left panel, red trace). This impairment of the tVOR affected most the sustained eye velocity during steady-state motion; in contrast, the earliest portion of the response changed less, and the oscillations of eye velocity were still present. The tVOR deficit was partially corrected when the target remained present; thus, visual tracking helped to compensate for the deficient tVOR (Figure 1C, right panel). Compensation of a deficient tVOR by pursuit has also been reported in a human with cerebellar agenesis [24]. Also similar to humans with disorders of the cerebellum [5], the decrease in the sustained tVOR in our monkeys could not be explained by impaired convergence [22].

### Modeling the interaural tVOR and the effect of Nod/Uv lesions: impaired integration of head acceleration

The disproportionate impairment of steady-state eye velocity after the Nod/Uv lesions suggested that there might be two components of the normal response, one related to head acceleration and the other to head velocity, and that the cerebellar contribution to these two components might differ. To test this hypothesis, we used a least-squares optimization technique to fit recorded eye velocity to a linear combination of head velocity and acceleration:
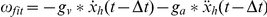
where ω_fit_ is the calculated eye velocity, 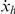 is head velocity, 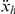 is head acceleration, and Δt is the time delay, i.e., the response latency relative to the chair feedback signal. For the fit, the latency was varied from 10 to 80 ms by 2 ms intervals (the sampling period of the chair signal was 2 ms). For each latency value, values of g_v_ (the velocity parameter) and g_a_ (the acceleration parameter) were determined by robust least-squares linear regression. For the series of possible latencies, the fit with the lowest squared residual error was considered to be the best.

**Figure 2:**
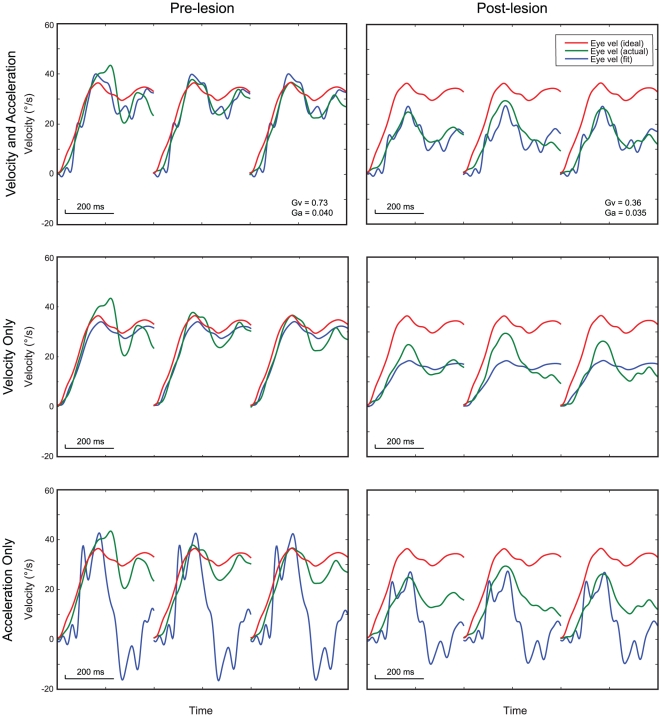
Example model fits for rightward translation (leftward eye velocity) in M2 (70 cm viewing distance) before and after the Nod/Uv lesion. Each panel shows three consecutive trials (first 400 ms of each) and depicts ideal horizontal eye velocity (red), measured eye velocity (green), and eye velocity calculated from the fit (blue). Note that the fit parameters were determined using data from all similar trials, not just these three. The top row shows the results of the full fit (head velocity and acceleration), the middle row the results of fitting to head velocity alone, and the bottom row the results of fitting to head acceleration alone. Only the fit that included both head velocity and acceleration modeled eye velocity well. For the post-lesion data, the velocity parameter dropped by about half, while the acceleration parameter changed little.

Figure 2 shows a representative fit from M2. Eye velocity was fit well by the combination of head acceleration and velocity but fit poorly to head acceleration or velocity alone. After the lesion, there was little change in the coefficient of the acceleration term, whereas the velocity coefficient dropped by about half.

Similar fits were performed for each monkey and for each of the two viewing distances, before and after the Nod/Uv lesions. For each of these eight conditions (M1/M2, near/far, pre/post), data were fit separately for each of the two eyes and the two directions of movement. The ratio of post-lesion to pre-lesion parameters was calculated, and the four values were averaged. For both monkeys, and at both viewing distances, there was a large drop in the velocity coefficient (g_v_) but not in the acceleration coefficient (g_a_). Overall, g_v_ dropped by 55+/−9.7% (p<0.002, t-test for ratio = 1) and g_a_ did not change (ratio 1.1, p>0.18).

**Figure 3:**
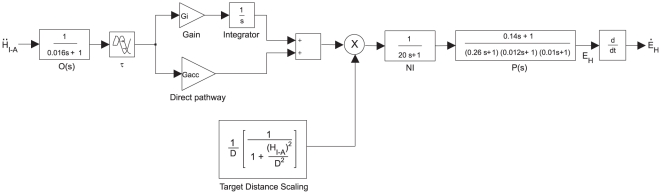
Simulink model of interaural TVOR. 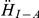
 = linear head acceleration. E_H_ = horizontal angular eye position. O(s) = otolith transfer function. G_i_ = TVOR integrator gain. G_acc_ = direct-pathway (acceleration) gain. τ = time delay (response latency). NI = velocity-to-position neural integrator. P(s) = eye plant, H_I−A_ = linear head position, D = perpendicular target distance.

**Figure 4:**
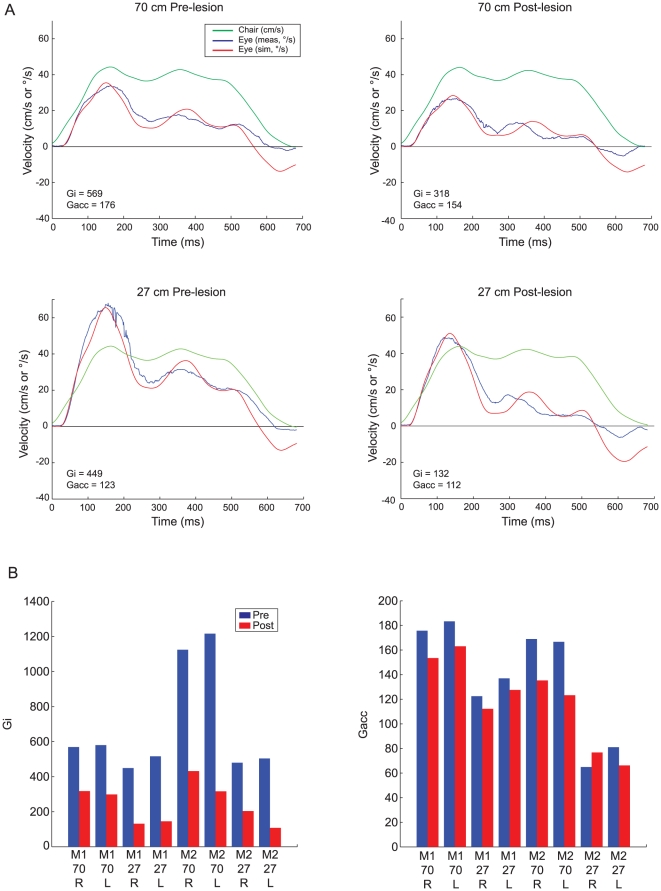
Simulink model optimizations. **a**. Results from M1 for leftward translation in darkness at each distance before and after Nod/Uv lesions. Leftward head velocity for one trial is shown in green. The blue trace represents the median rightward slow-phase eye velocity (saccades excluded) for all trials. The red trace is the simulated eye velocity using the parameter values determined by the optimization procedure. Note that each optimization used the whole series of trials, not the median eye velocity. **b**. Optimized values for integrator gain (G_i_) and acceleration gain (G_acc_) before (blue) and after (red) the Nod/Uv lesions, by animal (M1, M2), viewing distance in cm (27, 70), and direction of motion (R, L).

Based on this finding, we implemented a more complete model of the tVOR in Simulink® that incorporated a head-acceleration-to-velocity integrator as well as the dynamics of the otoliths and ocular plant (Figure 3). For the otolith transfer function, we used the first order approximation of other models [25], and for the plant we used the third-order transfer function of Fuchs, et al. [26]. The free parameters in the fitting process were the latency, represented by a time delay; the gain of the “direct” acceleration pathway (G_acc_); and the gain of the integrated pathway (G_i_). Optimization was performed using a Levenberg-Marquardt algorithm. The latency parameter was bounded (0–75 ms); the gains were required only to be positive. The error function was the difference between measured and simulated horizontal eye velocity for the whole series of trials. Again, we excluded quick phases and saccades based on criteria for eye acceleration and jerk. The results of the simulations (Figure 4) were in close accord with the findings of the first model: the integrator gain, G_i_, decreased by 62.2±8.6% (mean ±95% C.I., p<10^−5^), while the acceleration gain, G_acc_, decreased by only 12.6±7.8% (p<0.01). The latencies were not different (p>0.85).

**Figure 5:**
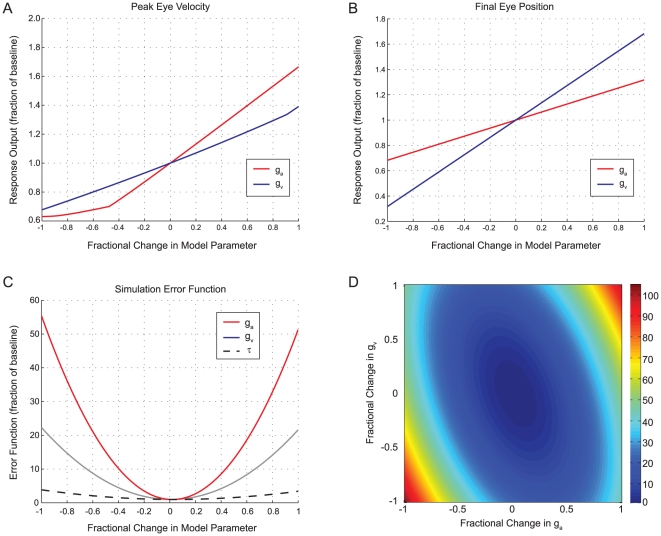
Sensitivity analysis of the Simulink model in **Figure 3**. **a**. Effect of varying the velocity (g_v_) and acceleration (g_a_) parameters on the peak of simulated eye velocity magnitude for a step of translation. The change in each parameter is represented as a fractional change from −1 (100% decrease, parameter set to zero) to 1 (100% increase, parameter value doubled). The dependent variable is the ratio of the new peak velocity to the baseline value when both parameters were at their optimized levels. The effect of varying the time delay is not shown; this does not affect response magnitude, but only shifts it in time. **b**. Effect of varying model parameters on final simulated eye position. **c**. Effect of varying model parameters on the error function of the simulation (the summed squared difference of simulated and actual eye velocities). Similar to **a** and **b**, the error function is shown as a percent of the baseline; thus, a value of 50 corresponds to an error magnitude that is 50 times greater. **d**. Effect on error function of simultaneous variation of g_a_ and g_v_.

To assess the robustness of the model fits, we performed a basic sensitivity analysis (Figure 5), in which we determined the effect of varying each of the model parameters on the model response (both peak simulated eye velocity and final eye position) and the error function, for a single step of translation. Not surprisingly, peak eye velocity was more sensitive to the acceleration parameter, and final eye position was more sensitive to the velocity parameter. The simulated error was affected by both the velocity and acceleration parameters, although more sensitive to the latter. There was less of an effect on the error of varying the time delay (latency). A two-dimensional analysis (Figure 5D) showed that the error function was sensitive to the values of the two parameters independently and that the values determined by the fitting process represented a true minimum over this parameter space.

Our model included the ‘common’ velocity-to-position neural integrator (NI), since the output of the model is eye position. Without the NI, the final eccentric eye position could not be maintained at the end of the head movement. Models of the saccadic system include a parallel non-integrated pathway to carry the saccadic pulse signal from the burst neurons directly to the ocular motor neurons. Since the burst neurons are not thought to contribute to the VORs, we omitted the direct pathway, similar to models of the rotational VOR. Nonetheless, we did test the effect of including it, which would have given eye velocity a component proportional to head jerk. When the direct pathway was included, the optimization procedure chose a gain that was very low. As Green and Angelaki have suggested [27], an alternative to our formulation would be to maintain the direct pathway around the NI but eliminate the direct pathway (acceleration pathway) parallel to the tVOR integrator. Although this could be equivalent in modeling the system's normal behavior, it would not account for the effects of the Nod/Uv lesions in our animals (see Discussion). Finally, we also found that the dynamics of the plant model were important. When we substituted the first-order approximation used in some prior tVOR models [25], [27], the data were fit poorly (see Discussion).

In our model, we modeled the tVOR acceleration-to-velocity integrator with a pure integrator, which represented the data well. We also tested the effect on the simulated eye movement of replacing this with a slightly leaky integrator, perhaps more biologically plausible. There was a small effect (<2% decrease) on final eye position (at 500 ms), as long as the time constant of this integrator was at least 5 s.

Overall, our model fit the recorded data well, but it did not fit eye velocity at the end of the trials, when head deceleration caused the simulated eye velocity to reverse direction. Such an anti-compensatory eye velocity was seen in the actual data, but the magnitude was much smaller. This suggests that the real tVOR has an additional nonlinearity that treats acceleration and deceleration differently. This might allow the eyes to “catch up” to the target when the head first accelerates but keep the eyes from going too far off target when the head stops moving. Similar properties are reported for smooth pursuit in which the transition dynamics differ between starting and stopping [28]. This is an example of the similarity between the tVOR and visual tracking mechanisms, such as smooth pursuit and short-latency ocular following, that stabilize on the fovea images of objects in a single depth plane, while ignoring objects at different distances. These eye movements function in a complementary way, much as do the rVOR and optokinetic nystagmus [29].

## Discussion

In this study we have shown that the cerebellar nodulus and uvula play an important role in the interaural tVOR: specifically, they contribute to the mathematical integration of head acceleration signals. We will discuss our findings in light of their implications for the role of the cerebellum in the tVOR and with respect to prior studies and models of the tVOR.

### Responses in normal animals

Most investigations of the interaural tVOR have used sinusoidal translations, although there have been several studies in monkeys and humans of responses to abrupt transient motion [30]–[34]. Consistent with these prior studies, we recorded a robust tVOR that was modulated by viewing distance and enhanced by a visual target.

### Role of the nodulus and uvula in the tVOR

The main effect of the Nod/Uv lesions on the interaural tVOR was to reduce sustained eye velocity during steady-state translation. When eye velocity was modeled as a linear combination of head acceleration and velocity, the effect of the lesions was to reduce the velocity but not the acceleration component. This finding suggests that the nodulus and uvula contribute to the integration of linear head acceleration signals derived from utricular afferents.

An important role for the nodulus and uvula in the tVOR is not surprising, given the dense projection of primary otolith afferents to this area [35]. That it is involved in an integration process is also reasonable, since the velocity-storage integrator of the rVOR [36] is also under control of the nodulus and uvula [14], [18], [37]. Moreover, a recent study found that Purkinje cells in the Nod/Uv have simple spike responses that appear to encode signals intermediate between head acceleration and velocity [21], which would be expected, based upon our lesion study and simulations, if these neurons participate in the acceleration-to-velocity integration.

Here we have considered the contribution of the Nod/Uv in the temporal domain as an integrator of head acceleration. Our post-lesion data are simulated well by a reduction in the scalar gain of this integrator. In the frequency domain, a parallel integrator acts to boost the response gain at low frequencies, and a reduction in integrator gain would therefore disproportionally reduce the low-frequency response of the reflex. Such an enhancement of the low-frequency tVOR is reflected in the frequency response of Nod/Uv neurons [21].

The enhancement of the rVOR at low frequencies has also been attributed to a process of integration, the so-called velocity-storage integrator. Could rotational velocity storage and the tVOR acceleration integrator share a common integration network? In fact, although both depend on the Nod/Uv, key differences in the signal processing requirements and the effect of Nod/Uv lesions suggest that these integrators are, at least to some degree, distinct. In particular, our data suggest that the nodulus and uvula *facilitate* integration for the tVOR, since lesions here impair it. On the other hand, the nodulus and uvula *inhibit* the integration that underlies angular velocity storage; they are responsible for the reduction in rVOR time constant that occurs during habituation [14].

Finally, the tVOR integrator may play an important role in tVOR motor plasticity. In an adaptation study, Zhou, et al. [38], reported that when the tVOR gain is high, eye velocity correlates well with head velocity. In contrast, when the tVOR gain is low, eye velocity correlates with head acceleration. The authors concluded that tVOR adaptation is likely to involve the linear acceleration-to-velocity integrator and they suggested that the cerebellum is a likely site for this plasticity. Our data support a central role for the Nod/Uv in this process and predict that Nod/Uv lesions would interfere with tVOR plasticity.

### Relationship to current models of the tVOR

Prior models of the tVOR have largely been based on the experimental data of Paige and colleagues [39], [40] and Angelaki [41] and have focused on explaining the high-pass frequency response, the effects of viewing distance and mechanisms by which the brain distinguishes head tilts from translational accelerations. We will discuss elements of these models as they apply directly to our experimental findings and, via direct simulations, we will compare them to our proposed model.

Most pertinent to our current study is the way in which these prior models have approached the “double-integration” question: how is the head acceleration signal carried by utricular afferents converted to eye position? Paige and colleagues [39], [40] included two integration steps, the common velocity-to-position neural integrator, and a second, leaky tVOR integrator (time constant 250 ms) in series with a high-pass filter (time constant 50 ms).

The model of Green and Galiana [27] included only a single central integrator. They attributed the second integration to the eye plant, which was represented by a simple first-order model with the same dynamics as the tVOR integrator of Telford, et al. [40], a fact that may explain the similar frequency responses of these two models. Angelaki, et al. [25], extended this model to incorporate known cell types in the vestibular nuclei and added first-order otolith afferent dynamics. Subsequent modifications of this model focused on interactions between canal and otolith signals for distinguishing head tilt from translation but largely kept the same dynamic properties [42], [43].

Musallam and Tomlinson [44] also proposed a model without an explicit second integrator. Instead they included a direct pathway for head acceleration signals to reach the ocular motor neurons. They pointed out that acceleration is in phase with head position and thus, if appropriately weighted, could approximate a position signal to the eye muscles. For transient stimuli, such as those used in our study, however, head acceleration can no longer be used to represent a position signal. Musallam and Tomlinson [45] later addressed this issue by recording the responses of secondary vestibular neurons to linear motion transients. During translations in the excitatory direction of a given neuron, the response of vestibular-only neurons reflected linear head velocity as well as acceleration, providing evidence of an integrated head acceleration signal at this level. The authors proposed that this behavior could be a direct consequence of membrane dynamics, but their findings are not inconsistent with our conclusion that the cerebellum contributes to this integration.

In a recent study, Green and Angelaki [46] revisited the question of integration for the tVOR by comparing the responses of burst-tonic neurons in the nucleus prepositus hypoglossi and medial vestibular nucleus (the presumed output of the velocity-to-position neural integrator) to head rotations and translations. The dynamics of the neuronal responses were similar with respect to both angular and linear head velocity. From this, they concluded that there must, in fact, be a second central integration step for the tVOR. Our experimental results and mathematical model of the tVOR are in accord with this suggestion.

To compare our experimental results and simulations to the predictions of other tVOR models, we implemented three of these prior models in Simulink: 1) Telford, et al. [40], 2) Green and Galiana [27], 3) Angelaki, et al. [25]. The model of Musallam and Tomlinson [44] was not easily assessed in Simulink, due to the fractional exponents in the transfer function representing the otolith afferents. For each of these we used the model structure provided in the respective manuscript with the parameters chosen by the authors. We did not alter the dynamics of any of these models, although we did adjust the overall scale of the output to match peak eye velocities, e.g., to take into account the response scaling based on target distance.

**Figure 6:**
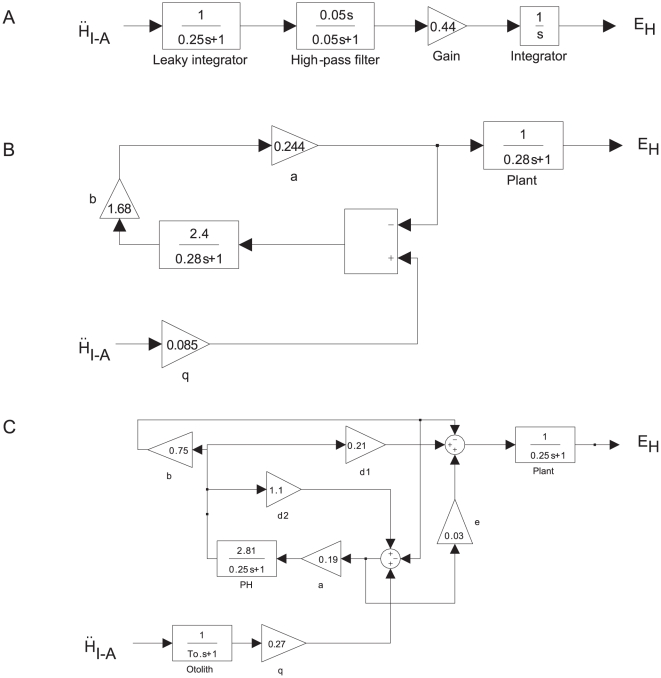
Schematics of Simulink representations of the three previous tVOR models whose behavior we compared to our own: a. Telford, et al. [**40**], b. Green and Galiana [**27**], c. Angelaki, et al. [**25**]. In each case, we used the parameters specified by the author (we did not fit the models to our data), but we scaled the output to match the peak simulated eye velocities and we matched the delay to that used in our model (31 ms). The input to each model was linear head acceleration from one of our steps of translation. As published, the Angelaki, et al., model (C) also included an input from the horizontal semicircular canal, which we omitted here, as we are considering only pure translational motion.

**Figure 7:**
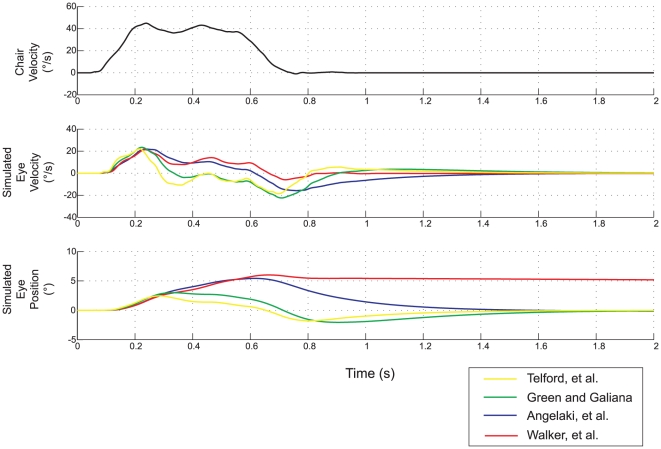
Simulation results for each of the four models. The top panel shows the linear head velocity profile of one of our experimental trials, which was used as the input for each model. The second two panels show simulated eye velocity and position (model outputs).

Schematics of the original models as we implemented them in Simulink are shown in Figure 6. For each of these models, we used as the input the linear acceleration profile for a single step of rightward translation from our experimental data. Figure 7 shows the simulated horizontal eye positions and velocities, including about 1.2 seconds following the end of the movement. The responses from all models are similar during the initial head acceleration but diverge substantially during the subsequent period of sustained translation. None of the models maintains eye velocity as well as the model we propose here (red trace), although the Angelaki, et al. [25], model comes closest.

What key features distinguish these models and determine their distinct dynamics? Only our model has two nearly pure integration steps: the tVOR integrator is a pure integrator and the velocity-to-position neural integrator has the usual time constant of 20 seconds, and thus this model best maintains eye velocity during sustained translation. The Angelaki, et al. [25], model comes closest, but without a true double-integration, it is unable to maintain eye position at its new position after the translation; within several hundred milliseconds, the eyes return to the zero position.

**Figure 8:**
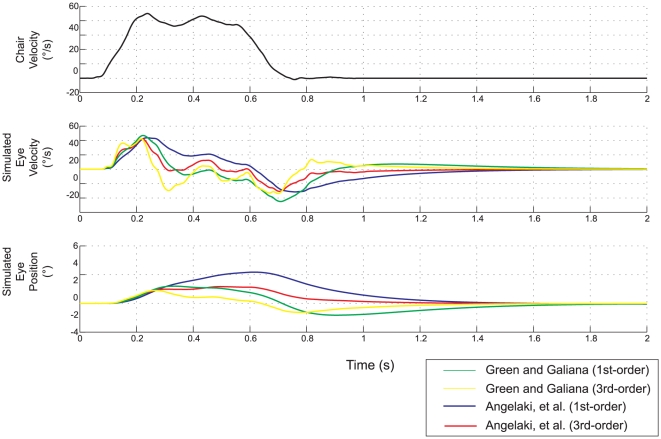
Effect of choice of eye plant model on the simulated eye movement. The top panel shows the head velocity profile of the input. The bottom two panels compare simulated eye velocity and position using the simple first order plant (time constant 0.25 ms) and the third-order plant.

The Green / Galiana [27] and Angelaki, et al. [25], models both incorporate a first-order model of the ocular plant with a similar time constant (0.26–0.28 seconds). As these authors point out, the low-pass characteristic of this plant model partially substitutes for the missing second integrator. This can be seen in Figure 8, which compares simulated eye velocities from each of these two models using the first-order and third-order plant models. Note the marked dependence of response dynamics on the plant model. The Telford, et al. [40], model did not include plant dynamics, but includes a leaky integrator on the input side with nearly the same dynamics. Hence the simulated eye velocity is very similar to the Green/Galiana [27] model (compare green and yellow traces in Figure 7).

**Figure 9:**
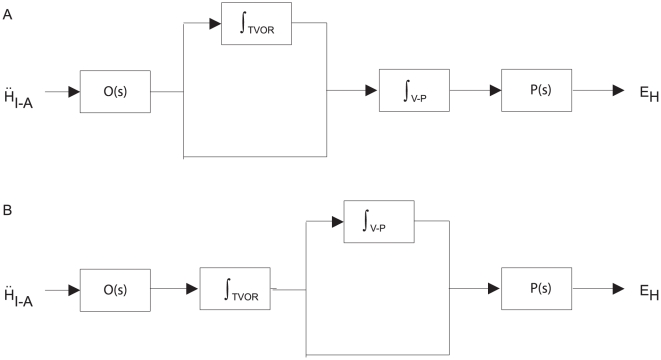
Comparison of basic model structure proposed here (a) with that suggested by Green and Angelaki [**46**], shown in b. **a**. In our model, the tVOR integrator is placed in parallel with a direct projection; the integrator is responsible for the component of eye velocity that is proportional to head-velocity, and the direct pathway provides the component that is proportional to head acceleration. The gain of the tVOR integrator is reduced by the Nod/Uv lesions, whereas the gain of the direct pathway is little affected. **b**. In the Green and Angelaki model, the direct pathway parallels the common integrator, formulated as the “plant compensator.” The tVOR integrator is incorporated into a “pre-filtering” stage. This model could not easily explain our post-lesion data. If the gain of the tVOR integrator were reduced, then both the velocity- and acceleration-dependent components of the response would be equally affected. On the other hand, reducing the gain of the common integrator could selectively alter the velocity-dependent component, but this would also cause pulse-step mismatch of saccades. It is unlikely that both integrators have a parallel direct pathway, because this would give eye velocity a component that is proportional to head jerk.

Two other studies have posited a tVOR integrator [45], [46], but in neither case was an explicit model proposed, and thus we were not able to compare them directly with our model or to simulate their behavior in response to our experimental stimulus. Green and Angelaki [46] speculated, based on neural recordings of burst-tonic neurons in the nucleus prepositus hypoglossi and similar to the model of Telford, et al. [40], that the head acceleration signal is integrated in a “prefiltration” stage and then passed to the common velocity-to-position neural integrator in parallel with a direct pathway (analogous to the pulse-step integrator of the saccadic system). In a sense, this would be similar to our model, except that the order of the integrators is effectively reversed – the second integrator has the parallel non-integrated pathway (Figure 9).

Our results do not eliminate the possibility of a parallel direct pathway to the common neural integrator (as suggested by the modeling of Cannon and Robinson [47] and proposed by Green and Angelaki [46]). On the other hand, our lesion data do require that the tVOR integrator have a parallel pathway; otherwise, it would not have been possible for our lesions to affect differentially the velocity and acceleration components. The alternative would be for the Nod/Uv lesions to decrease the gain of the common neural integrator, leaving its parallel pathway untouched. It is unlikely, however, that the effect of our lesions was on the velocity-to-position integrator, as this would also be expected to cause gaze-holding deficits and post-saccadic drift. These deficits have been associated with lesions of the flocculus and paraflocculus [48], but we did not observe them in our monkeys with Nod/Uv lesions. Thus, it appears that there are two separate functional integrators: 1) a tVOR integrator, under control of the Nod/Uv, and 2) the common neural integrator, under control of the floccular complex.

**Figure 10:**
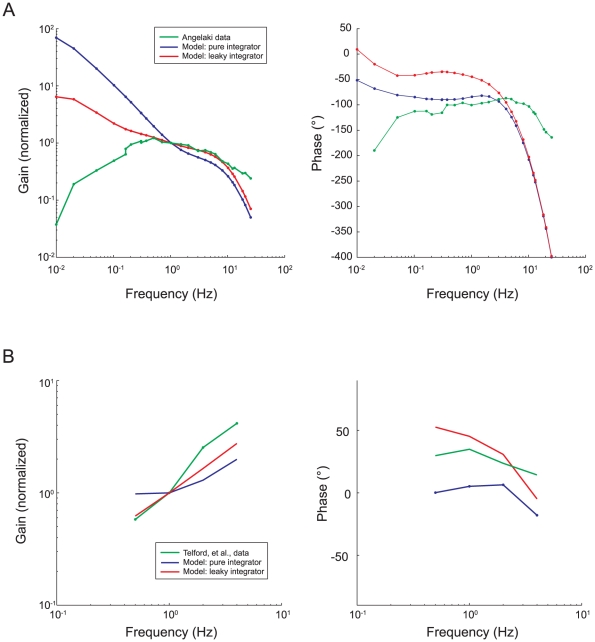
Frequency response simulations of the Simulink model, compared to published data. Data values from the two studies were extracted from published figures (as noted), using a public-domain program (G3Data). Model simulations were run for inputs at each of the frequencies of the respective data set. The amplitude and phase of the model output were computed using a sinusoidal fitting technique [54]. To account for differences in absolute scaling (e.g., different effective fixation distances), the amplitudes of both the simulated and actual data were normalized to give a response gain of unity at 1 Hz. In each case, the green trace shows the actual data extracted from the published figure, the blue trace shows the model response when the tVOR integrator is representated by a pure integrator, and the red trace the model response with a leaky integrator (τ = 10 s). **a**. Comparison of model responses to data of Angelaki [see Figure 9 of reference 41]. Consistent with the published data, response amplitudes are presented as the ratio of eye velocity to head acceleration (equal amplitude head accelerations were used as the model input in this case), and response phases similarly represent the phase of eye velocity relative to head acceleration. Phases differ from the published data by 180°, because 180° was added to the measured data by Angelaki for illustration purposes (see figure legend). **b**. Comparison of model responses to data of Telford, et al. [see Figure 12 of reference 40]. In this case, response gains and phases are determined relative to translational head velocity.

For technical reasons, we were not able to test the responses to sinusoidal stimulation in our monkeys. We did, however, compare the predictions of our model to the data from the previous studies of Angelaki [41] and Telford, et al. [40]. The results are shown in Figure 10. In the mid-frequency range, likely to represent the primary operating range of the tVOR, the model predictions were similar to the actual data. The main differences are that our model predicts a higher gain at the lowest frequencies, due to the presence of the additional integrator, and it predicts a lower amplitude and a greater phase shift at very high frequencies, compared to the Angelaki data. Further investigation will be necessary to reconcile these differences. Possibilities include the ways in which the experiments had to be performed (both very low and very high frequency stimulations are subject to technical limitations) and the existence of nonlinearities at the high frequency extremes as, for example, has been suggested by Minor and colleagues [49] or adaptation operators, as has been proposed for very low frequency responses.

To summarize, these simulations provide several important insights into how information must be processed for the tVOR. First, only a model that truly incorporates a double mathematical integration is able to account for our data. The leaky integration included explicitly in the Telford, et al. [40], model, or in the form of the first-order eye plant in the Green/Galiana [27] and Angelaki, et al. [25], models, does not suffice. In these models, eye position could not be held in its new position, after a step of translation. To accomplish this, a much longer time-constant is required for the tVOR integrator. Second, replacing the first-order plant model with a more realistic third-order model makes the need for a second integration even more apparent. Third, the parallel integrator and direct pathways must be distinct from the common velocity-to-position integrator.

Our results and simulations here point to a central, mathematically explicit role for the cerebellar nodulus and uvula in processing of information to compensate for translation of the head. They further emphasize that the signal processing capabilities of the cerebellum, in particular mathematical integration, are tailored to the specific requirements for optimal motor control. This signal processing is able to take into account the wide range of sensory signals the cerebellum receives and the specific mechanical complexity of the effector organs that must be moved.

## Materials and Methods

### Ethics Statement

All animals in this study were handled in strict accordance with good animal practices as defined by the relevant national standards, including the NIH Guide for the Care and Use of Laboratory Animals. The research protocol, including all study procedures, was approved by the Johns Hopkins University's Animal Care and Use Committee, for which the U.S. Public Health Service Office of Laboratory Animal Welfare Animal Welfare Assurance Number is A3272-01. The animals were monitored closely throughout the course of the study by the university veterinary service. Animals were housed as a group in the same room in full view of other animals, and frequent enrichment was provided through toys and a variety of food treats and food puzzles. Surgical procedures were performed in a dedicated animal operating suite using full aseptic technique, barbiturate or inhalation anesthesia, and cardiorespiratory monitoring. Post-operative analgesia was provided with buprenorphine, beginning before recovery from anesthesia and continuing with scheduled dosing while monitoring animals closely for any signs of discomfort. During periods in which experiments were conducted, daily weights ensured adequate fluid and nutritional status.

### General procedures

We studied two (one female and one male) juvenile rhesus monkeys (4–6 kg) before and after surgical lesions of the inferior vermis. All procedures were approved by the Animal Care and Use Committee of the Johns Hopkins Medical Institutions. Eye movements were recorded using binocular dual scleral search coils, in a three-field coil system. During experiments, the animal was seated in a primate chair with the head immobilized, and the chair was secured firmly to a linear sled (Acutronic, Switzerland). The coil frame was attached tightly to the top of the primate chair, such that the head was centered within it, and it remained fixed with respect to the head when the chair moved. Eye coil signals were demodulated by frequency detectors, sampled at 1000 Hz, and stored for later analysis. A belt-driven motor moved the chair along the interaural axis, under computer control. Chair position was measured by a linear transducer (Inductosyn®, Ruhle Companies, Inc.) and sampled at 500 Hz.

Before these experiments, one of the monkeys underwent intracranial trochlear nerve section (M1, right trochlear nerve), followed by a right inferior oblique recession and a left inferior rectus recession, as part of a different study [50]. Data collection for the present study commenced 38 days following the last eye muscle surgery.

Cerebellar lesions were performed by one of the authors (R.J.T.) using standard neurosurgical procedures, as previously described [51]. For both animals, behavioral experiments began on the eighth post-operative day. The final recording was an additional 35 days later for M1 and 21 days later for M2. We did not observe any recovery of the tVOR over the period of post-operative recording; thus, as for pre-operative recordings, all data were combined for the analysis. As previously reported, post-mortem histological sections confirmed removal of the entire nodulus and the majority of the uvula in both animals [see Figure 1 of reference 51]. In M2 only, the lesion also included portions of lobule VII and VIII.

### Experimental Paradigm

Each trial began with the chair still and the monkey fixating a laser target, back-projected on a translucent screen that was either 27 or 70 cm in front of the eyes. Other than the target, the room was dark. The target was either straight ahead or 10° up or down from the center. We did not observe any difference in the dynamics of horizontal eye velocity based on vertical eye position. Thus, for the purpose of this study, we combined trials from all three vertical positions, in order to maximize the amount of data available for each optimization procedure.

After the monkey achieved fixation of the target (determined by a fixation window), the chair was accelerated (0.26 *g*) to a speed of 40 cm/s, then moved at constant speed for 320 ms, after which it was decelerated to a stop (0.20 *g*). The total displacement of each trial was 20 cm. Leftward and rightward trials were alternated (i.e., motion direction was not randomized). The fixation target either remained on or was extinguished at the onset of chair motion; in the latter case, motion occurred in complete darkness.

### Data Analysis

Data were analyzed using custom programs written in MATLAB™ and in Python, using the numpy, scipy (www.scipy.org) and matplotlib packages (matplotlib.sourceforge.net). Raw data were converted to three-dimensional rotation vectors, eye position vectors, and angular velocity vectors using methods that have been previously described [53]. The reference positions were obtained during monocular fixation of the target at center position. According to the right-hand rule, leftward and downward positions and velocities are positive.

Similar trials (same animal, eye, motion direction, target distance, and target condition) were combined for analysis. Trials were synchronized on the time that the chair speed crossed a threshold of 1 cm/s.

Ideal eye velocity is defined as the eye velocity that would keep the fovea on the target. Because an eye rotation compensates for a head translation, ideal eye velocity for the tVOR depends on the distance from the eye to the target. The exact relationship is:
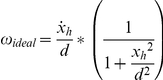
where ω_ideal_ is ideal eye velocity, x_h_ is the linear position of the head, 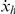 is linear head velocity, and d is the perpendicular distance from the eye to the target.

### Models

First, eye velocity was fit to a simple model based on a linear combination of head acceleration and velocity. Details of the model are given in the Results. Saccades and quick phases were detected using an algorithm based on eye acceleration and jerk [54] with threshold criteria of 1400°/s^2^ and 50,000°/s^3^, respectively. These segments were excluded when calculating eye velocity (Figure 1) and when fitting the data to the model. For each fit, a robust least-squares linear regression was performed (function rlm in R). Only trials from translation in the dark were included in the fit, and a single fit was performed for all trials from a given monkey, target distance, eye, time (before or after surgery), and motion direction. To account for response latency, in each case we performed the fit over a range of time delays (10 ms to 80 ms in 2 ms steps, based on the chair position sampling frequency of 500 Hz). We selected the fit with the lowest squared residual error.

For the Simulink model (Figure 3), we added transfer functions from previously published models (see Results) to represent the dynamics of otolith afferent responses and the ocular motor plant. We also included the ocular motor eye-velocity to position integrator. We used Levenberg-Marquardt optimization (MATLAB function lsqnonlin) to fit the same data used for the prior fits. There were three free parameters in the optimization: time delay, acceleration gain (G_acc_), and integrator gain (G_i_). The error function was the difference between recorded and simulated eye velocity for the set of trials. Additional details of this model are given in the Results section and corresponding figures.
